# British Pharmaceutical Conference

**Published:** 1920-08-14

**Authors:** 


### British Pharmaceutical Conference.

### THE LIVERPOOL GATHERING.

This annual gathering of British pharmacists was ended on July 22 at Liverpool, and attracted a large gathering, which included prominent institutional pharmacists. A reception was held by the Lord Mayor of Liverpool at the Town Hall on Monday. Tuesday and Wednesday were devoted to the reading of scientific and business papers, whilst an admirable series of excursions, including a visit to an Atlantic liner and a river trip was arranged for the ladies. The conference concluded with a motor trip to the North Wales district.

The President of the conference, Mr. C. A. Hill, B.Sc., in his address upon the present position and future prospects of pharmacy, stated that if British manufacturers were to capture and hold the fine chemical trade more generous help would be required from the Government—especially in regard to concessions of duty-free alcohol for manufacturing, which obtained in Germany and other countries. Mr. Hill also deplored the lack of facilities for the testing of preparations clinically in Great Britain, pointing out that we were behind other countries in this respect.

A presentation was made to Mr. Horace Finnemore, B.Sc., until lately Chief Pharmacist at Guy's Hospital, in recognition of his services as secretary to the conference for many years. Mr. C. Hampshire (University College Hospital) has now taken over the post of hon. secretary to the conference in succession to Mr. Finnemore.

